# Academic detailing and adherence to guidelines for Group B streptococci prenatal screening: a randomized controlled trial

**DOI:** 10.1186/1471-2393-13-68

**Published:** 2013-03-19

**Authors:** Jussara M Silva, Airton T Stein, Holger J Schünemann, Ronaldo Bordin, Ricardo Kuchenbecker, Maria de Lourdes Drachler

**Affiliations:** 1 Department of Epidemiology, Federal University of Rio Grande do Sul, Porto Alegre, Brazil; 2 Department of Epidemiology, Federal University of Health Sciences (UFCSPA) and Ulbra, Porto Alegre, Brazil; 3 Clinical Epidemiology & Biostatistics Department and Department of Medicine, McMaster University, Hamilton, Canada; 4 Department of Epidemiology, School of Medicine, Federal University of Rio Grande do Sul, Porto Alegre, Brazil; 5 School of Medicine, Federal University of Rio Grande do Sul, Hospital das Clinicas of Porto Alegre, Porto Alegre, Brazil; 6 Postgraduate Programme in Collective Health, School of Nursing, Federal University of Rio Grande do Sul, Porto Alegre, Brazil

**Keywords:** Guidelines, Physicians, Pregnancy, Screening, Streptococci

## Abstract

**Background:**

Clinical practice guidelines (CPGs) recommend universal prenatal screening for Group B *Streptococcus* (GBS) to identify candidates for intrapartum antibiotic prophylaxis to prevent early onset neonatal GBS infection. Interventions to promote physician adherence to these guidelines are imperative. This study examined the effectiveness of academic detailing (AD) of obstetricians, compared with CPG mailshot and no intervention, on the screening of pregnant women for GBS.

**Methods:**

A randomized controlled clinical trial was conducted in the medical cooperative of Porto Alegre, Brazil. All obstetricians who assisted in a delivery covered by private health insurance managed by the cooperative in the 3 months preceding the study (*n* = 241) were invited to participate. The obstetricians were randomized to three groups: direct mail (DM, *n* = 76), AD (*n* = 76) and control (C, *n* = 89, no intervention). Those in the DM group were sent guidelines on GBS. The AD group received the guidelines and an educational visit detailing the guidelines, which was conducted by a trained physician. Data on obstetrician age, gender, time since graduation, whether patients received GBS screening during pregnancy, and obstetricians who requested screening were collected for all participant obstetricians for 3 months before and after the intervention, using database from the private health insurance information system.

**Results:**

Three months post-intervention, the data showed that the proportion of pregnant women screened for GBS was higher in the AD group (25.4%) than in the DM (15.9%) and C (17.7%) groups (*P* = 0.023). Similar results emerged when the three groups were taken as a cluster (pregnant women and their obstetricians), but the difference was not statistically significant (Poisson regression, *P* = 0.108). Additionally, when vaginal deliveries were analyzed separately, the proportion screened was higher in the AD group (75%) than in the DM group (41.9%) and the C group (30.4%) (chi-square, *P* < 0.001).

**Conclusions:**

The results suggest that AD increased the prevalence of GBS screening in pregnant women in this population.

## Background

Group B *Streptococcus* (GBS) infection is the most common bacterial infection transmitted vertically from mother to child during labor and delivery. The neonatal infection is a leading cause of neonatal morbidity and mortality and can severely affect the quality of life of the child in the short and long term. Efforts are needed internationally to prevent neonatal GBS infections.

Medical guidelines on prenatal care strongly recommend GBS screening of the vagina and anus between 35 and 37 weeks of gestation and intrapartum antibiotic prophylaxis of colonized women to prevent neonatal infection [1,2]. Despite their success as a strategy to prevent perinatal GBS disease, these recommendations have mostly been applied in developed countries only [3]. In Brazil, a prevalence of maternal colonization by GBS of between 15 and 25% suggests that universal screening is likely to be cost-effective in this country [4,5], and GBS screening is recommended by the Brazilian Medical Guidelines on prenatal care [2]. Interventions potentially effective in promoting the adherence of obstetricians to these guidelines are lacking in this country.

Internationally, the implementation of medical guidelines is a challenge that usually takes many years [6,7]. Passive continuing medical education, or traditional education, appears to be of limited effect in guideline implementation. Studies have shown that educating physicians in their office is a promising strategy for changing medical practice [8,9], mainly through academic detailing (AD), an intervention that combines interactive, one-on-one communication conducted by trained healthcare professionals—typically pharmacists, physicians or nurses—with evidence-based, noncommercial information. AD has been successfully applied to increase the adherence of health professionals to guidelines for screening [10,11] and to decrease inappropriate use of medicines [9]. It involves face-to-face education, with additional elements such as educational materials, educational meetings, or audit and feedback [12].

Some studies have assessed the effectiveness of AD in the implementation of guidelines in obstetrics [13], with mixed effects [13] and favorable results [14]. Multifaceted behavioral interventions, including AD visits, appeared to increase the prophylactic use of oxytocin during the third stage of labor and reduced the likelihood of episiotomy in one study [15].

No research has investigated the effect of AD in promoting GBS screening in the private or public sector in Brazil. This study examined the effectiveness of AD, conducted through an educational visit by a trained physician to obstetricians, in promoting screening for vaginal and rectal GBS colonization.

## Methods

A randomized controlled clinical trial of interventions to promote prenatal screening for GBS was conducted. The participant service was a major private health insurance company managed by a medical cooperative in Porto Alegre, South Brazil [16]. All obstetricians (*n* = 241) who had provided outpatient prenatal care and assisted in at least one delivery (vaginal or cesarean section) covered by this health insurance in the 3 months before the study (April to June, 2008) were invited to take part. The same doctors were involved in prenatal care. The participant obstetricians were allocated randomly into three groups: direct mail (DM, *n* = 76), AD (*n* = 76) and control (C, *n* = 89). A list of random numbers generated by the Statistical Package for the Social Sciences (SPSS Inc., Chicago, IL, USA) was used to allocate the obstetricians.

The DM intervention comprised printed guidelines on antenatal GBS screening sent by post to the obstetrician’s private office in July, 2008. The AD intervention included these guidelines and a 30-minute face-to-face education-oriented interview on antenatal GBS screening based on the national guidelines for antenatal care [2]. This was conducted by a trained physician and took place in the private offices of the participating obstetricians, in July and August, 2008. No intervention was provided to the C group.

The electronic database of the participating organization was used to gather the following information about the obstetricians and their performance of antenatal care and deliveries both 3 months pre-intervention (April to June, 2008) and 3 months post-intervention: obstetrician age, gender, year of graduation in medicine, number of years of antenatal clinical practice in the medical cooperative, number of births assisted, and the frequency of GBS screening requested.

Multivariable Poisson regression analysis was used to investigate the effect of the intervention on the proportion of pregnant women tested for GBS, controlling for potential confounding factors (physician age and sex, time since graduation in medicine and number of years of antenatal clinical practice in the medical cooperative). A *P* value of 0.05 or less was considered statistically significant.

The study was approved by the Ethics Committee of Federal University of Rio Grande do Sul, protocol number 2007792.

## Results

There were 908 deliveries during the baseline assessment period. There was no evidence of a difference between the three groups of obstetricians (AD, DM and C) in relation to the baseline variables: age, gender, number of years since graduation in medicine, number of years of antenatal clinical practice in the medical cooperative, proportion of obstetricians requesting a GBS screening, and proportion of pregnant women tested for GBS (15.8%, 17.0% and 21.4%, respectively) (Table 1).

**Table 1:** Sociodemographic data of obstetricians and details of their practice before the intervention (n = 241)

	**AD**^**a**^	**DM**	**C**	**p-value**
	**N=76**	**N=76**	**N=89**	
**Age** (in years), mean ^b^	45.6±8.6	45.7±8.0	46.1±8.0	*p*=0.90
**Female, **% ^c^	75.0	63.2	62.9	*p*=0.251
**Time elapsed since graduation in medicine** (in years), mean ^b^	21.2±8.5	21.2±7.7	21.1±8.1	*p=*0.99
**Time in healthcare plan** (in years), mean ^b^	15.5±8.1	15.7±6.6	16.3±7.2	*p=*0.84
**Obstetricians who had requested GBS screening pre-intervention **^d^, % ^c^	31.6	26.3	37.1	*p=*0.33
**Pregnant women tested for GBS pre-intervention **^d^, n and (%) ^c^	38/240 (15.8)	59/346 (17.05)	69/322 (21.42)	*p=*0.19

^**a**^ AD: Academic detailing; DM: direct mail; C: control group; Data are presented as ^b^ mean ± standard deviation, ^c^ numbers (percentage); ^d^ Group B streptococcus (GBS) screening in the 3 months before the intervention.

There were 849 deliveries during the 3-month post-intervention period. In this time, 58, 67 and 78 obstetricians in the AD, DM and C groups, respectively, performed at least one delivery. The other 38 obstetricians did not assist with a delivery in this period.

In the post-intervention period, there was evidence of a difference between the three groups in the proportion of pregnant women tested for GBS (chi-square, *P* = 0.023); the proportion was higher for the AD group (25.4%) than for the DM group (15.9%) and the C group (17.2%) (Table 2).

**Table 2:** Requests for and performance of GBS testing in pregnant women for 3 months post-intervention

	**AD**^**a**^	**DM**	**C**	***Chi-square p-value***
	**N=76**	**N=76**	**N=89**	
**Obstetricians**				
Who requested GBS^b^ screening post intervention^c^	28 (36.8)	21 (27.6)	32 (30.0)	0.412
**Pregnant**				
Proportion of pregnant women tested for GBS post intervention^d^	52/205 (25.36)	45/283 (15.90)	64/361 (17.72)	0.023

^a^ AD: Academic detailing; DM: direct mail; C: control group; ^b^ Group B streptococcus- one or more cultures; ^c^ Data are presented as numbers (percentage); ^d^ Number of pregnant women screened for GBS divided by the number of women who gave birth in the period.

Additionally, when the three groups were considered as a cluster (pregnant women and their obstetricians), the frequency of GBS screening was higher for the AD group than for the C group, but this difference was not statistically significant (Poisson regression, *P* = 0.108) (Table 3).

**Table 3:** Comparison between the intervention groups and control group on application and performance of GBS testing in pregnant women, considering the cluster “pregnant women and their obstetricians”

**Requested culture GBS**	**Prevalence rate**	**95% CI**^**a**^	***p***
**AD**^**b **^**group**	1.43	0.92-2.21	0.108
**DM**^**b **^**group**	0.89	0.53-1.51	0.683
**C**^**b**^	Reference		

^a^ confidence interval; ^b^ AD: Academic detailing; DM: direct mail; C: control group.

Similarly, when vaginal deliveries were analyzed separately, the proportion of GBS screening was higher in the AD group (75%) than in the DM group (41.9%) and the C group (30.4%) (chi-square, *P* < 0.001) (Table 4).

**Table 4:** Effect of interventions on requests for and performance of culture for Group B streptococci in women with vaginal deliveries

**Groups**	**N**^**o**^**Vaginal deliveries**	**N**^**o**^**GBS**^**a **^**screening**	**% GBS**^**a **^**screening**
**Academic detailing**	32	24	75
**Direct mail**	43	18	41.9
**Control**	56	17	30.4
**Total**	**131**	**59**	**45.0**

^a^Group B streptococci; Chi-square 2dF, *p* < 0.001.

## Discussion

This paper reports a randomized controlled clinical trial of an intervention implemented in the context of usual prenatal outpatient care. The trial showed that pregnant women cared for by obstetricians who received an AD-based intervention were more likely to be screened for GBS than were those cared for by obstetricians who received printed guidelines only or no intervention. Similar results emerged when the three groups were taken as a cluster (pregnant women and their obstetricians), but the difference was not statistically significant, maybe because of low study power. The study was based on obstetrician members of a medical cooperative and the number of obstetricians and patients was relatively small. There was also loss of participant obstetricians on intention-to-treat analysis because 38 obstetricians did not conduct deliveries during the study period.

The AD intervention was associated with a significant increase of 9.5% in the frequency of prenatal GBS screenings compared with the passive printed material distribution or the no intervention scenario. This modest result for AD is in line with similar studies on guideline implementation [17].

In the present study, when vaginal deliveries were analyzed separately, the proportion of screening was higher in the AD group (75%) than in the DM group (41.9%) and the C group (30.4%). These results suggest that the AD intervention was particularly relevant in women who had a vaginal delivery, for which prenatal GBS screening is the most useful in preventing neonatal infection.

The trial was conducted with obstetricians who had assisted a delivery paid for by the medical cooperative in the 3 months before the intervention, regardless of whether the obstetrician had requested GBS screening. The sample size was insufficient to analyze the effect of the intervention separately for obstetricians who had and had not previously requested screening. Because some studies have shown no impact of untargeted outreach visits [18,19], further studies should investigate the effect of AD on GBS screening separately for these two groups. Outreach visits may also face barriers in the form of resistance to change [20], which should be assessed in future studies. Factors that most discourage the use of AD are time spent in the office for continuing medical education, physicians’ perception of wasting working time in the office to receive AD and continuing medical education provided by a non-physician [21]. The attitudes of the obstetricians were not assessed in the present study.

Another limitation of this trial is the relatively short follow-up of 3 months, which may have led to overestimation of the observed benefit of the intervention. Additionally, there is a possibility of contamination of the DM and C groups, but underestimation of the effect of AD is unlikely because the obstetricians worked in private medical outpatient practices and had relatively little interaction with each other.

In this trial, the educational visits were conducted by a trained physician, in line with a previous study that showed that visits made by peers tend to be more effective for behaviors related to collaboration with others and practice organization, compared with interventions conducted by non-peers [22]. However, interventions provided to obstetricians by other health professionals, such as nurses, should be examined in other studies.

This study has the potential to contribute to best practice, showing that a brief intervention based on AD in medical practice may have a significant impact in increasing the number of patients screened for GBS. This study is also relevant to obstetric practice in middle income economies such as Brazil where a significant proportion of the population receives care paid through private health insurance.

Several factors may prevent obstetricians following prenatal screening policy. Among these is the fact that there is a high rate of cesarean section in private clinics in Brazil (e.g. 83.3% in the present study), which may prompt obstetricians to consider the promotion of prenatal GSB screening unnecessary, especially for women already scheduled to undergo elective cesarean section. While the World Health Organization recommends a maximum of 15% cesarean sections among total births, Brazil has one of the highest cesarean section rates in the world, with a national average of 43%, reaching 80% in the private healthcare setting [23]. Although this is strongly related to higher social class, the main determinant of the elevated rate of cesarean section is delivery in a private maternity unit [24,25], as the sample studied here shows. The main reasons given by obstetricians are the convenience of programmed intervention for the obstetrician, uncertainty regarding the possibility of hypoxia or fetal trauma, and lack of preparation of the woman for the birth [26,27].

The high cesarean section rate in the organization studied here may have played a role in the observed low impact of AD, as suggested by the finding that AD had a greater effect when women who underwent vaginal delivery were analyzed separately. The obstetrician’s decision to perform a cesarean section may have influenced his or her decision on whether to perform GBS screening because the aim of identifying women harboring GBS is to prevent neonatal colonization during vaginal delivery and not during cesarean section.

From the perspective of clinical practice in low resource settings, there may be gaps between the scientific evidence for an intervention and its adoption in clinical practice [28], including a lack of financial and non-financial resources to implement changes in healthcare. The low remuneration of medical care is likely to contribute to the high rate of caesarean sections in Brazil, especially in the private health sector, whereas vaginal delivery requires more working hours and lower remuneration proportionally. Financial incentives may be effective in changing healthcare professional practice [29]. Although GBS testing is an easy and affordable screening method, the adoption of this practice is likely to be affected by the use of elective cesarean section. Further studies are needed to establish whether opting for a cesarean is a barrier to GBS screening. Advances in behavioral economics are driving efforts to use material or financial incentives to overcome economic obstacles or a lack of effective motivation, and recipients are incentivized to engage in health-related behaviors or practices with which they are already familiar and that they regard as beneficial or worthwhile.

## Conclusions

In conclusion, a benefit was achieved by the AD intervention in this study, in that more pregnant women were screened for GBS. Further longer term studies are needed before the benefits of AD in promoting the adoption of evidence-based guidelines on prenatal care are fully understood.

### Consent

Written informed consent was obtained from all participant obstetricians for publication of this report.

## Competing interests

JM Silva is an Advisor to the Cooperative, a member of the Commission for Support and Guidance for implementation of Clinical Guidelines. The authors declare that they have no competing interests.

## Authors’ contributions

JMS - designed the overall study and conducted the analysis and drafted the manuscript. ATS - designed the overall study conducted the analysis and drafted the manuscript. HJS - conducted the analysis and drafted the manuscript. RB - conducted the analysis and drafted the manuscript. RK - conducted the analysis and drafted the manuscript. MLD - conducted the analysis and drafted the manuscript. All authors read and approved the final manuscript.

## Pre-publication history

The pre-publication history for this paper can be accessed here:

http://www.biomedcentral.com/1471-2393/13/68/prepub
